# Small Molecule Screen Reveals Joint Regulation of Stress Granule Formation and Lipid Droplet Biogenesis

**DOI:** 10.3389/fcell.2020.606111

**Published:** 2021-04-16

**Authors:** Triana Amen, Daniel Kaganovich

**Affiliations:** ^1^Department of Experimental Neurodegeneration, University Medical Center Göttingen, Göttingen, Germany; ^2^1Base Pharmaceuticals, Boston, MA, United States

**Keywords:** stress granule, lipid droplet, PPAR, lipid metabolism, fatty acids

## Abstract

Metabolic regulation is a necessary component of all stress response pathways, because all different mechanisms of stress-adaptation place high-energy demands on the cell. Mechanisms that integrate diverse stress response pathways with their metabolic components are therefore of great interest, but few are known. We show that stress granule (SG) formation, a common adaptive response to a variety of stresses, is reciprocally regulated by the pathways inducing lipid droplet accumulation. Inability to upregulate lipid droplets reduces stress granule formation. Stress granule formation in turn drives lipid droplet clustering and fatty acid accumulation. Our findings reveal a novel connection between stress response pathways and new modifiers of stress granule formation.

## Introduction

Lipid droplets (LD) are ubiquitous lipid storage organelles that are involved in regulating energy homeostasis and membrane synthesis. Their importance to the cell has been thought to derive from the need to sequester excess fatty acids, as an energy reserve and to prevent lipotoxicity (Guo et al., 2009; Nguyen and Olzmann, 2017; Walther et al., 2017; Olzmann and Carvalho, 2019). Recently, however, novel unanticipated roles in stress response and protein folding have been proposed for LDs (Guo et al., 2009; Li et al., 2012; Moldavski et al., 2015; Bischof et al., 2017; Nguyen et al., 2017). Concurrently, a link between LD homeostasis and neurodegenerative disorders is beginning to emerge (Velazquez and Graef, 2016; Onal et al., 2017; Pennetta and Welte, 2018). These new findings clearly indicate that LDs have a stress response role and make it less surprising that many stresses, including pH changes, oxidative stress, mitochondrial perturbations, endoplasmic reticulum stress response, and autophagy activation all lead to LD formation (Boren and Brindle, 2012; Li et al., 2012; Krahmer et al., 2013; Rambold et al., 2015; Henne et al., 2018; Jin et al., 2018; Petan et al., 2018). Although some of the molecular mechanisms governing LD biogenesis are beginning to emerge (Gubern et al., 2009; Li et al., 2012; Jin et al., 2018; Olzmann and Carvalho, 2019; VandeKopple et al., 2019), how these mechanisms are activated by stress signals is not fully understood. There is therefore a pressing need to determine the mode of communication between a stress signal, conventional stress responses, and lipid stress response.

In normal conditions, cells sense excess fatty acids by activating peroxisome proliferator-activated receptor (PPAR)-mediated transcription, which leads, among other things, to LD biogenesis (Dalen et al., 2004; Varga et al., 2011; Rohwedder et al., 2014; Gorga et al., 2017; Kim et al., 2017). PPAR nuclear receptors are activated by various ligands including fatty acids themselves (Dalen et al., 2004; Rodriguez and Kersten, 2017). The PPAR response encompasses genes involved in lipid trafficking, fatty acid-binding proteins, fatty acid oxidation, and LD structural proteins [e.g., perilipins (PLINs)] (Poulsen et al., 2012). The PPAR family consists of three members: PPARα, PPARδ (also called PPARβ), and PPARγ (Rodriguez and Kersten, 2017). PPARα ensures energy availability during fasting and starvation by upregulating lipid storage and fatty acid oxidation (Rakhshandehroo et al., 2010). PPARγ is a master regulator of adipogenesis in mammalian cells (Rosen and Spiegelman, 2001; Contreras et al., 2013). Interestingly, PPAR activation antagonizes mammalian target of rapamycin (mTOR) complex during fasting, leading to its inhibition (Barak et al., 1999). mTOR is a general regulator of translation, and its inhibition leads to translation downregulation (Sengupta et al., 2010). It is therefore perplexing that the PPAR-regulated transcription overrides the global translation inactivation that results from mTOR inhibition. An important piece of the puzzle seems to be the activation of stress granule (SG) formation by stress-induced mTOR inhibition (Fournier et al., 2013; Jevtov et al., 2015; Sabatini, 2017; Sfakianos et al., 2018). SGs are multifunctional membraneless organelles, with an important role in managing translation during stress, including recruitment of mTOR (Takahara and Maeda, 2012; Fournier et al., 2013; Thedieck et al., 2013). SGs appear during various stresses that curiously overlap with those that induce LD formation (Kedersha et al., 2005; Buchan and Parker, 2009; Sabatini, 2017). Interestingly, SG proteins are associated with lipid droplets during hepatitis C infection (Buchan et al., 2011), and SG component DDX3 is directly involved in lipid metabolism (Ariumi et al., 2011; Tsai et al., 2017). Thus, there is a mechanistic connection between SG formation and LD biogenesis, both of which can result from mTOR inactivation (Li et al., 2012; Fournier et al., 2013).

Here, we demonstrate a positive feedback relationship between SG and LD formation. We show that induction of stress granules leads to the formation of LDs and that inability to mount a LD response leads to inability to form SGs. Our data identify several novel compounds that simultaneously trigger SG formation and fatty acid accumulation in LDs. Overall, our study provides evidence of a joint regulation of SG and LD biogenesis.

## Results

### Small Molecule Screen Identifies the Correlation Between Stress Granule and Lipid Droplet Formation

**Figure 1:**
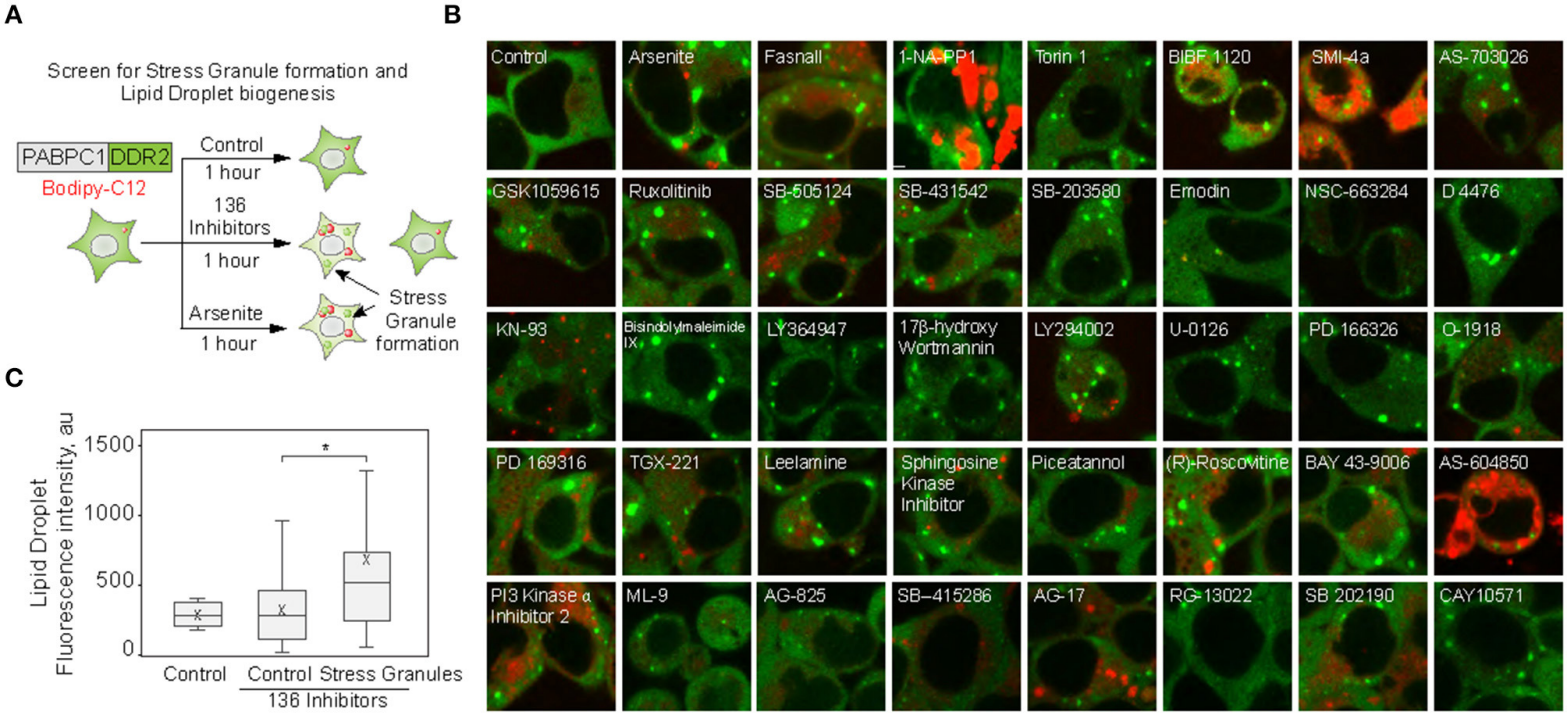
Small molecule screen identifies the correlation between stress granule and lipid droplet formation. **(A–C)** Inhibitor screen for fatty acid accumulation and SG formation. Cells expressing PABPC1-DDR2 were seeded on a 96-well glass-bottom plates and grown to 80–90% confluency. One hundred thirty-six inhibitors (100 μM) were added to the media for 1 h, LD dye (Bodipy-C12, Red, 1 μM) was added 30 min prior to the experiment. Cells were visualized by confocal microscopy; inclusion formation and LD accumulation were assessed. Quantification **(C)** shows fluorescence intensity in lipid droplets, box indicate 25–75 percentiles, cross indicates mean, whiskers are 9–91 percentiles, for control sample (no inhibitors added), control screen samples (samples without inclusions, *n* = 98), stress granule samples (samples with inclusions, *n* = 38). Representative confocal images of the samples with inclusions are shown **(B)**; refer to Supplementary Figure 1 for extended screen results. *p < 0.05.

We set to explore the role of SGs in promoting LD biogenesis. Using an endogenously tagged PABPC1 cell line, we screened 136 small molecule inhibitors for the ability to induce LD formation, also scoring SG assembly (Figure 1A; Supplementary Figure 1) (Shih et al., 2012; Amen and Kaganovich, 2020a). Thirty-eight molecules induced PABPC1-positive inclusion formation (Figure 1B). The majority of the molecules that we examined are kinase inhibitors, which explains the high proportion of positive hits due to a known role of SGs in cellular signaling (Kedersha et al., 2013; Sfakianos et al., 2018; Heberle et al., 2019; Amen and Kaganovich, 2020). Next, we analyzed the upregulation of LD by calculating the accumulation of LD dye (fluorescent C12-Bodipy) in the samples that formed SGs and in the samples that did not result in SG accumulation (Figures 1B,C). Despite variation (Supplementary Figure 1), SG-forming cells significantly upregulated LDs, while the rest of the treated cells were not different form an untreated control (Figures 1B,C; Supplementary Figure 1). Thus, SG formation can be used as a predictor of LD biogenesis.

### Inhibition of Stress Granule Formation Reduces Lipid Droplet Formation

**Figure 2:**
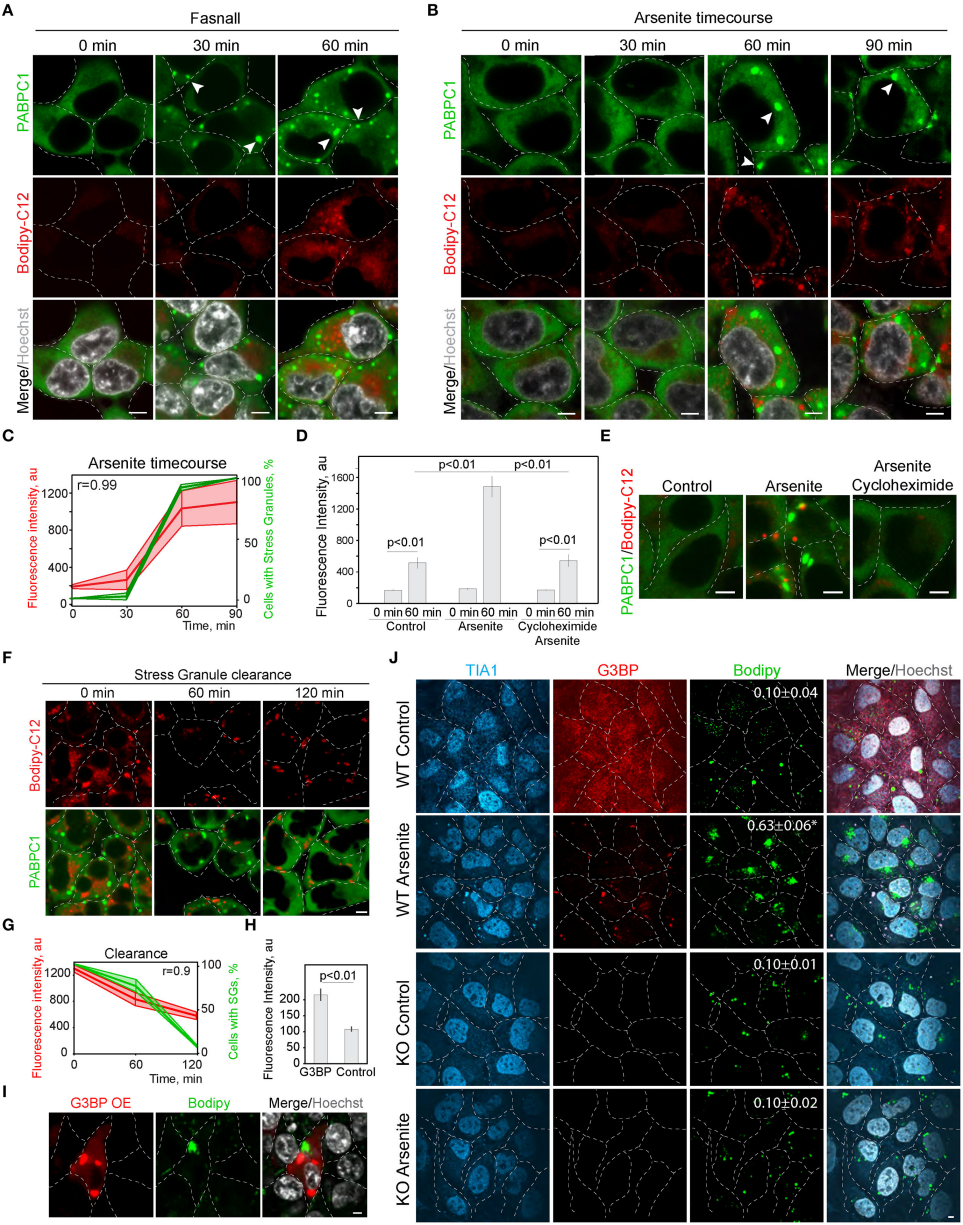
Inhibition of stress granule formation reduces lipid droplet formation. **(A)** SG formation timeline during Fasnall treatment showing fatty acid accumulation. Cells expressing CRISPR/Cas9-tagged PABPC1-DDR2 were incubated with Fasnall (100 μM) for indicated amounts of time, LD dye (Bodipy-C12, 1 μM) was added 30 min prior to the imaging together with Hoechst (10 μg/ml). Representative confocal images are shown. Arrows indicate SGs. Scale bar, 5 μm (see also, Supplementary Figure 2C). **(B)** SG formation timeline during sodium arsenite treatment showing LD accumulation. Cells expressing CRISPR/Cas9-tagged PABPC1-DDR2 were incubated with arsenite (100 μM) for indicated amounts of time; fatty acid dye (Bodipy-C12, 1 μM) was added 30 min prior to the imaging together with Hoechst (10 μg/ml). Arrows indicate SGs. Scale bar, 5 μm. **(C)** Quantification of SG formation and LD accumulation during arsenite treatment (corresponding to **B**). Graph shows percentage of cells with SGs in the population and LD fluorescence intensity, mean ± SD. Pearson correlation coefficient (*r*) is 0.99. **(D)** Quantification of fatty acid (Bodipy-C12) accumulation in LDs **(H)**. Cells expressing CRISPR/Cas9-tagged PABPC1-DDR2 were incubated with arsenite (100 μM) or arsenite and cycloheximide (10 μg/ml) for 90 min. Fatty acid dye (Bodipy-C12, Red, 1 μM) was added 30 min prior to the experiment. Graph represents fluorescence intensity of bodipy-tagged FAs in LDs, mean ± SEM, *n* = 30. **(E)** Disruption of SG formation results in the decrease in fatty acid accumulation. Cells expressing CRISPR/Cas9-tagged PABPC1-DDR2 were incubated with arsenite (100 μM) or arsenite and cycloheximide (10 μg/ml) for 90 min. Fatty acid dye (Bodipy-C12, 1 μM) was added 30 min prior to the imaging. Representative confocal planes are shown; scale bar, 5 μm. **(F)** SG clearance timeline after arsenite (200 μM) treatment showing LD clearance. Cells expressing CRISPR/Cas9-tagged PABPC1-DDR2 were incubated with arsenite for 90 min followed by washing it twice and incubating with a fresh medium for 120 min, LD dye (Bodipy-C12, 1 μM) was added 30 min prior to the imaging. Representative confocal images are shown. Arrows indicate SGs; Scale bar, 5 μm (see also Supplementary Figure 2C). **(G)** Quantification of SG formation and LD accumulation during arsenite recovery (Corresponding to **F**). Graph shows percentage of cells with SGs in the population and LD fluorescence intensity, mean ± SD. Pearson correlation coefficient (*r*) is 0.9. **(H,I)** Confocal microscopy **(I)** and quantification **(H)** of LD fluorescence intensity during G3BP overexpression. HEK293T cells transfected with RFP-G3BP were stained with Bodipy, 1 μM and Hoechst (10 μg/ml). Representative confocal image is shown. Refer to Supplementary Figure 2G. Graph represents fluorescence intensity of Bodipy in LDs, mean ± SEM, *n* = 30. **(J)** Confocal microscopy of LD in U2OS WT and G3BP1/2 KO cells during control and arsenite treatments. Quantification of LD clustering is shown in the LD frames (3d column).

In the screen (Figure 1; Supplementary Figure 1), we scored SG formation as a binary parameter (cells with and without SGs). Thus, we next analyzed the correlation of SG and LD formation during the treatment with known SG inducers (Kobayashi et al., 2012). We tracked LD formation with Bodipy-C12 in SG-forming conditions; it was clear that the appearance of SGs correlates directly with the pronounced growth of LDs (Figures 2A,C; Supplementary Figure 2A). Induction of SG, using arsenite treatment (Figures 2B,C), and induction of SG formation by Fasnall treatment (Kobayashi et al., 2012; Amen and Kaganovich, 2020,b) (Figure 2A; Supplementary Figure 2A), both led to a corresponding acceleration in LD biogenesis with a correlation coefficient of 0.99 for arsenite treatment. Conversely, the disruption of SG formation during arsenite treatment with cycloheximide greatly impaired LD biogenesis (Figures 2D,E), implying a dependence of LD proliferation on SGs and translation. So far, we scored LD formation using quantification of C12-Bodipy accumulation in LDs. However, Bodipy-C12, in addition to accumulating in LDs, localizes to the little specks fusing to LDs and membranes (Supplementary Figures 2B,C). We confirmed that Bodipy-C12 and Bodipy colocalize in LDs (Supplementary Figures 2B,C), and scored the number of LDs in cells during arsenite and arsenite with cycloheximide treatments using a neutral LD dye—Bodipy. We found a significant increase in LD number in cells forming SGs (Supplementary Figure 2D). Interestingly, inducing clearance of SGs by removing the arsenite from the media resulted in a decrease in LD content to almost the control levels (Figures 2F,G). Next, we triggered SG formation using G3BP overexpression (Supplementary Figure 2E) (Reineke et al., 2012; Takahara and Maeda, 2012; Alwarawrah et al., 2016) and scored LD intensity in cells with and without SGs. Induction of SGs resulted in an increased LD content (Figures 2H,I). Finally, we assessed how a genetic disruption of SG formation affects LD formation during stress. We scored LD formation using Bodipy staining during control and arsenite treatments in G3BP1/2 KO (Matsuki et al., 2013; Yang et al., 2020) and WT U2OS cells (Figure 2J). Arsenite treatment resulted in a significant increase in LD clustering (Figure 2J; Supplementary Figure 2F). We quantified clustering as a proportion of proximal to each other LDs using intensity profile overlapping. Interestingly, WT cells exhibited a higher number of smaller LDs, while G3BP1/2 KO cells contained on average larger LDs (Supplementary Figure 2G). Together, these data points toward the role of SGs in LD formation.

### PPAR Alpha Regulates SG Formation

**Figure 3:**
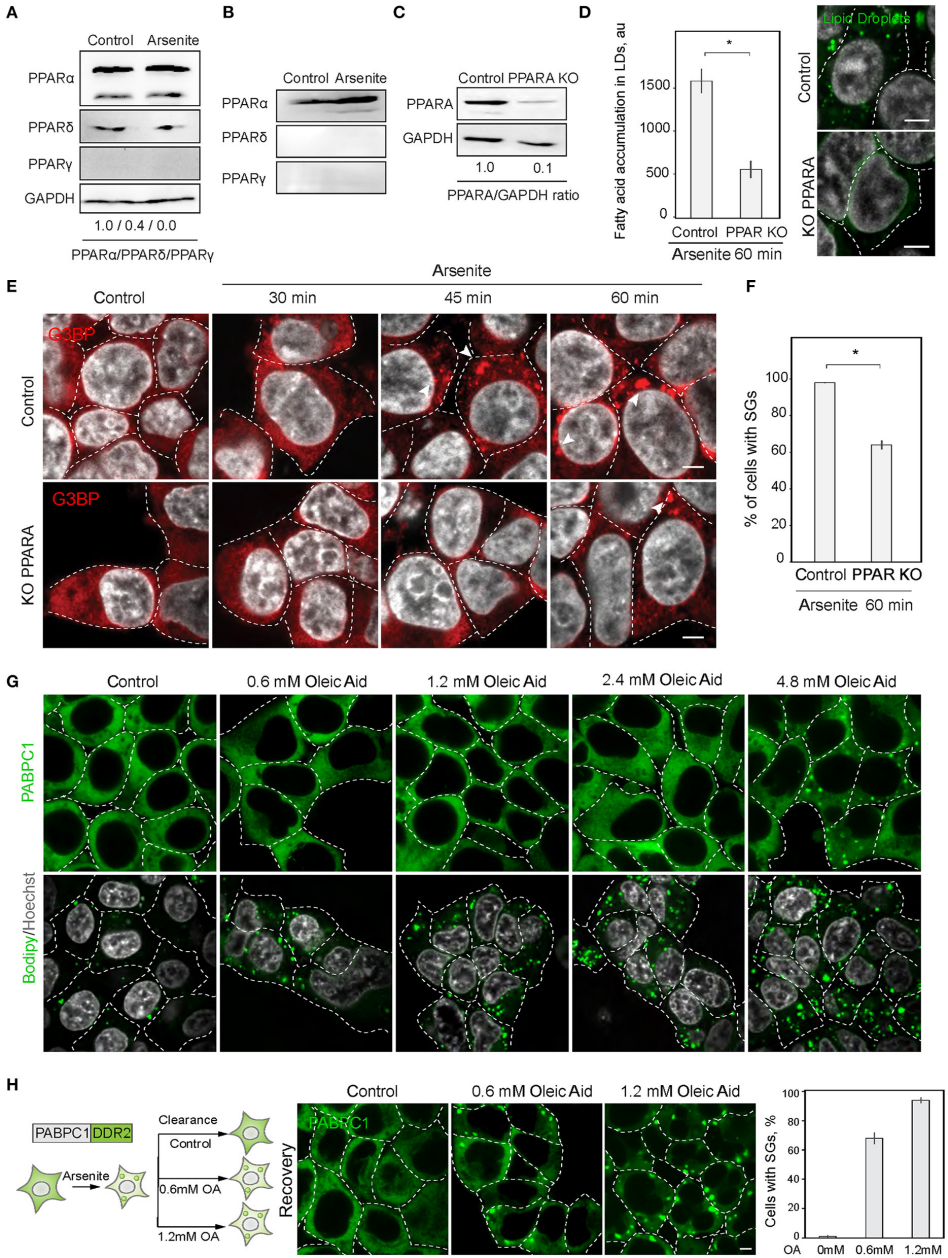
PPAR alpha regulates SG formation. **(A)** PPARA isoform is prominent in SH-SY5Y cells. Cells were grown to 90% confluency. Control and arsenite-treated (100 μM, 1 h) cells were lysed and analyzed by western blot. **(B)** PPARA isoform is prominent in HEK293 cells. Cells were grown to 90% confluency. Control and arsenite-treated (100 μM, 1 h) cells were lysed and proteins were analyzed by western blot. **(C)** Verification of partial PPARA knockout (KO). **(D)** PPARA regulates LD accumulation. Control and PPARA knockout cells were treated with arsenite (100 μM) for indicated amounts of time and fixed in 4% PFA. LDs were stained with Bodipy (green, 1 μM), Hoechst (10 μg/ml) was added 15 min prior to the imaging, and confocal planes are shown; scale bar, 5 μm. Graph fluorescence intensity of FAs in the LDs, mean ± SEM, *N* = 30 (in each condition). **(E,F)** PPARA regulates SG formation. Control and PPARA knockout cells were treated with arsenite (100 μM) for indicated amounts of time and fixed in 4% PFA. SG formation was analyzed with G3BP antibodies, Hoechst (10 μg/ml) was added 15 min prior to the imaging, and confocal planes are shown; scale bar, 5 μm. Graph represents percentage of SGs in the population, mean ± SEM, *N* = 30 (in each condition). **(G)** Confocal microscopy of SG and LD formation during oleic acid (OA) treatment. HEK293T cells were treated with the indicate amounts of OA for 1 h before the imaging. SG are visualized with an endogenous PABPC1-DDR2. LDs are stained with Bodipy. **(H)** Confocal microscopy of SG clearance in HEK293T PABPC1-DDR2 cells during 120 min recovery from 90 min arsenite treatment (200 μM) with and without the addition of indicated amounts of OA. Graph represents percentage of SGs in the population, mean ± SEM, *N* = 30 (in each condition). *p < 0.05.

Next, we explored the possibility of the reciprocal regulation of SG and LD formation. In order to upregulate lipid metabolism, cells mobilize a transcriptional response promoted by the PPAR nuclear receptor (Dalen et al., 2004). We therefore constructed a cell line with a disrupted PPAR receptor and LD upregulation. PPAR nuclear receptors have several isoforms with partially overlapping targets, PPARα, PPARδ (also called PPARβ), and PPARγ, which are differentially expressed in cells and tissues (Figures 3A,B). HEK293T and SH-SY5Y cells both express PPARα, while SH-SY5Y cells also express PPARγ (Figures 3A,B). To inhibit cellular LD response, we constructed a partial PPARα knockout in HEK293T cell line using CRISPR/Cas9 (Figure 3C). A 90% reduction of PPARα resulted in reduced ability to induce LD during stress (Figure 3D). Inability to upregulate LD response in the PPARα KO, correlated with a significant decrease in SG formation (Figures 3E,F). These data suggest that either PPARα regulates both SG and LD formation or LD response regulates SG formation. We assessed whether induction of LDs with oleic acid result in SG formation. Only a very high concentration of fatty acids in the media (4.8 mM) resulted in SG formation in 1 h of incubation (Figure 3G). In 24 h of incubation, there was a clear tendency of LDs to form clusters with increasing concentration of fatty acids in the media; however, cells that formed SGs did not survived (S3A). Thus, only sublethal concentrations of fatty acids induce SGs. Finally, we scored if clearance of SGs during recovery is affected by oleic acid in the media. Surprisingly even low amount of oleic acid drastically impaired SG clearance (Figure 3H). These data indicate that LD formation alone is not sufficient to induce SG formation.

### Reciprocal Regulation of Stress Granule Formation and Lipid Droplet Biogenesis Through mTOR and PPAR Activation

**Figure 4:**
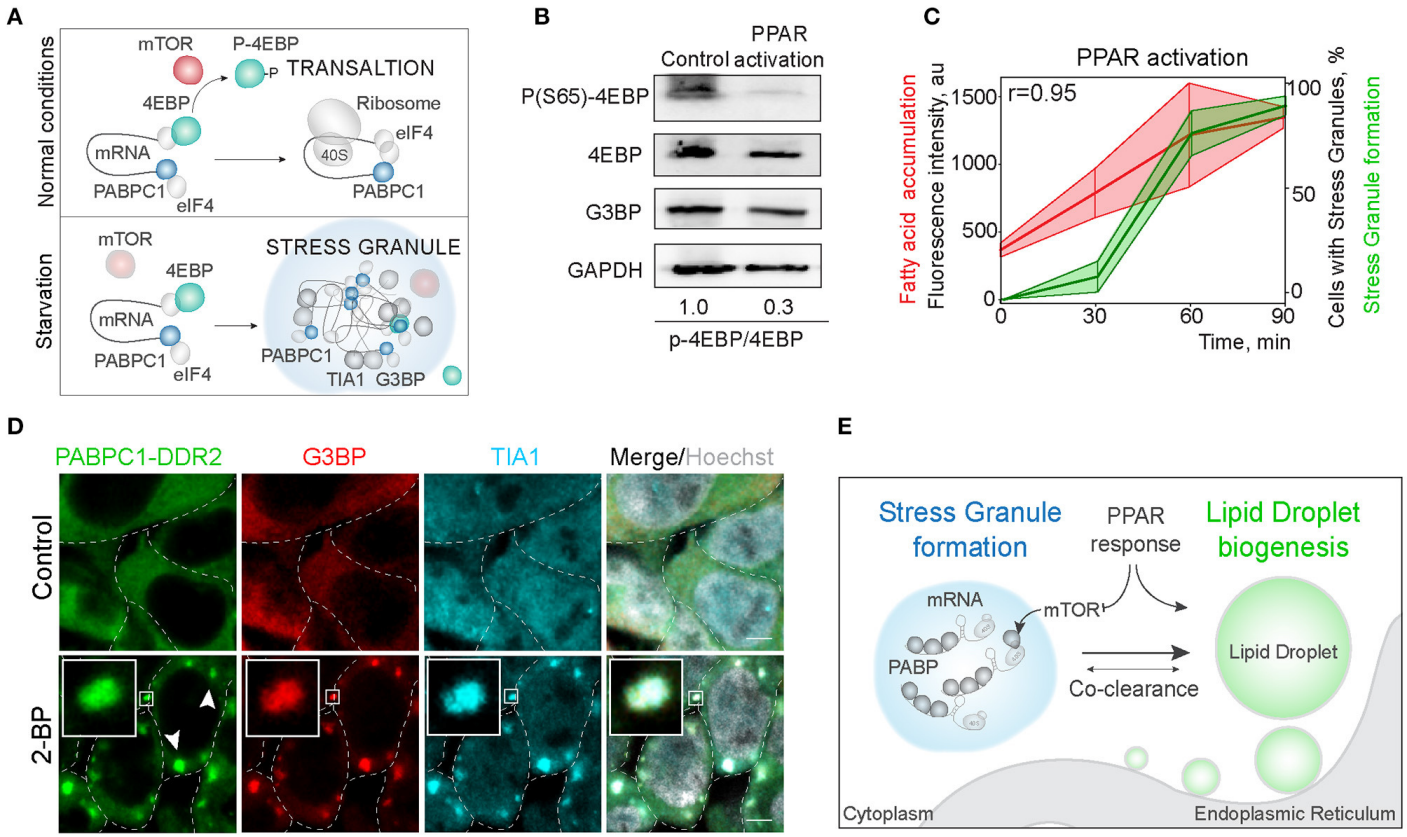
Activation of LD formation triggers SGs. **(A)** Schematic of mTOR regulation of translation favoring SG formation. During normal conditions, mTOR phosphorylates 4EBP allowing translation initiation. Downregulation of mTOR results in translation inhibition and stress granule formation. **(B)** PPAR activation leads to mTOR inactivation. Cells were incubated with PPAR activators (rosiglitazone, clofibrate, and GW501516, 100 μM) for 3 h. mTOR activity was assessed by 4EBP phosphorylation (Ser65) state. Ratio of phosphorylated to non-phosphorylated 4EBP is shown **(B)**. **(C)** Quantification of SG formation and LD accumulation during PPAR activation (refer to Figure 1A). Graph shows percentage of cells with SGs in the population and LD fluorescence intensity, mean ± SD. Pearson correlation coefficient (*r*) is 0.95. **(D)** Immunofluorescence showing SG markers localization to the inclusions. PABPC1-DDR2 cells were treated with 2-BP (200 μM) for 1 h. Cells were fixed and stained with anti-G3BP and anti-TIA1 antibodies. Confocal planes are shown; inlets show SGs; scale bar, 5 μm. **(D)** Model of the joint regulation of SG and LD formation.

Next, we explored the mechanisms of regulation of LD and SG formation. PPAR activation, that promotes lipid droplet biogenesis is a known inhibitor of mTOR kinase signaling (Yang et al., 2020). Significantly, mTORC1 is a constituent of SGs (Fournier et al., 2013; Thedieck et al., 2013). SG formation has been proposed to regulate mTOR activity by facilitating the inhibition of mTOR kinase, whereas mTOR signaling has been proposed to regulate SG formation by suppressing translation (Figure 4A) (Fournier et al., 2013; Thedieck et al., 2013; Jevtov et al., 2015; Sfakianos et al., 2018). We confirmed the mTOR inhibition during PPAR activation, using 4EBP phosphorylation, and in parallel-visualized SG formation during PPAR response (Barak et al., 1999; Teleman, 2005; San et al., 2015) (Figure 4B; Supplementary Figure 3B). Indeed, PPAR activation was sufficient to trigger mTOR inhibition (Figure 4B). Using PPAR agonists is sufficient to upregulate lipid accumulation and LD biogenesis (Gorga et al., 2017) (Figure 4C). Interestingly, activation of lipid droplet response, with a PPAR activator (2-bromopalmitate) (2-BP), triggered SG formation (Figures 4C,D; Supplementary Figure 3C). SG formation correlated with fatty acid accumulation during LD formation (Pearson correlation coefficient = 0.95) (Figure 4C). To confirm that PABPC1 inclusions are SG, we used common SG markers, G3BP and TIA1 (Figure 4D), and independent human cell lines (Supplementary Figure 3E). These data indicate that SG and LD formation are both regulated by the PPAR response, which can facilitate SG formation by inhibiting mTOR kinase, and LD formation can be a consequence of mTOR inhibition (Li et al., 2012) or other unknown pathways (Figure 4E).

## Discussion

During stress, cells activate distinct protective mechanisms such as upregulation of chaperones and protein quality control compartments, synthesis of antioxidant proteins, and increase in LD formation, allowing cells to mitigate the consequences of stress and adapt to stress conditions (Lindquist, 1981; Gingras et al., 1999; Kaganovich et al., 2008; Spriggs et al., 2010). To regulate these seemingly distant phenomena, cells reconfigure their translation patterns, often completely halting bulk translation and sequestering translation initiation complexes in SG compartments (Kedersha et al., 2005; Guil et al., 2006; Takahara and Maeda, 2012; Kaganovich, 2017; Amen and Kaganovich, 2020). SGs are also thought to serve as a signaling hubs, sequestering major kinases, like mTOR and PKC, which functions as a master switch for stress adaptation (Kaganovich et al., 2008; Kedersha et al., 2013; Amen and Kaganovich, 2015, 2020; Sfakianos et al., 2018; Heberle et al., 2019). We set out to mechanistically integrate seemingly distant stress response phenomena—SG formation and LD biogenesis.

To determine whether the SGs and LDs consistently form together in various stress conditions, we screened a library of 136 independent inhibitors for co-occurrence of SG formation and LD biogenesis. We found several novel compounds that induce SG formation; however, due to often exceeding the optimal inhibitory concentration (we used a uniform 100 μM concentrations), SG formation in many cases maybe unrelated to a specific effect of the inhibitor, as we demonstrated for Fasnall (Kobayashi et al., 2012). Strikingly, cells with SGs demonstrate significant upregulation of fatty acid accumulation in LDs, as compared with cells in conditions that did not form SGs. SG formation induced by arsenite or G3BP overexpression affects LD accumulation and inhibition of SG formation prevents accumulation and clustering of LDs. These data confirm that SG and LD formation are jointly an integral part of stress response. Interestingly, the SG component DDX3 has been shown to regulate lipid homeostasis (Ariumi et al., 2011). In addition, LDs are required for efficient clearance of membraneless compartments during stress (Guo et al., 2009), indicating that there may be additional functional connections between SGs and LDs. To explore these connections, we examined SG regulation by PPAR response (Barak et al., 1999; Dalen et al., 2004; Poulsen et al., 2012). PPAR is a nuclear receptor which, when activated, cooperate with RXR, binds to PPRE and facilitate translation of lipid metabolism effectors, including multiple fatty acid binding proteins (FABPs), and LDs components (PLINs) (Rodriguez and Kersten, 2017; Amen and Kaganovich, 2020). We found that pharmacological hyperactivation of PPAR led to SG formation and mTOR inhibition (Barak et al., 1999). Additionally, 90% reduction of the PPAR alpha isoform, whose activation was shown to inhibit the mTOR kinase (Rakhshandehroo et al., 2010), resulted in a reduction in SG formation. Even though PPAR response has a striking correlation with SG formation in this study, we only used a pharmacological hyperactivation or a knockout as regulators of Lipid Droplet biogenesis. Whether physiological activation of PPAR response drives SG formation or how different PPAR isoforms affect SG formation warrants a more rigorous study on this topic.

Additionally, we show that Stress Granule clearance correlates with the reduction of LD levels, and that presence of fatty acids in the media at a concentrations used to induce LDs (Michelet et al., 2018) significantly slows down SG clearance during stress recovery. Whether or not LDs are necessary for clearance of SGs, as it was demonstrated for Protein Quality Control inclusions (Guo et al., 2009) remains to be determined. What is clear, however, is that relationship between SGs and LDs maybe explained through signaling regulation. mTOR kinase has been shown to regulate SG formation (Fournier et al., 2013), thus we hypothesized that TOR inhibition by PPAR activation can trigger SG formation. There is evidence for reciprocity of the mTOR-PPAR interaction, hence inhibition of mTOR *via* SG formation results in the activation of PPAR response (Barak et al., 1999; Sfakianos et al., 2018). Thus, it is interesting to explore if SG formation can indeed facilitate the PPAR response. It is not clear how PPAR target translation evades bulk translational inhibition, which results in SG formation during mTOR inhibition (Kedersha and Anderson, 2007; Takahara and Maeda, 2012; Exner et al., 2019). It is possible that a recently demonstrated model of SGs sequestering longer mRNA plays a role (Sheikh and Fornace, 1999). Our study provides a novel avenue on exploring the role of SGs in the biogenesis of LDs. Given that there are physiological stress conditions, like starvation and infection (Barak et al., 1999; Buchan and Parker, 2009; Khong et al., 2017), that can result in both SG formation and LD biogenesis, it would be interesting to understand the role of cooperation of these pathways for cellular adaptation and stress response.

## Experimental Procedures

### Cell Culture and Cell Lines

HEK293T and U2OS (WT and G3BP1/2 KO) cells were maintained in DMEM supplemented with 10% fetal bovine serum (FBS), 1% penicillin/streptomycin, at 37°C/5% CO_2_, SH-SY5Y cells were maintained in 1:1 F12/DMEM media supplemented with 10% fetal bovine serum (FBS), 1% penicillin/streptomycin at 37°C/5% CO_2_. Cells modified *via* CRISPR/Cas9 were maintained as above with addition of puromycin (2 μg/ml, Sigma) during selection of clonal populations.

### Fatty Acid Import and Quantification in Lipid Droplets

Fatty acids (bodipy-C12, 1 μM) were added 30 min prior to quantification (Holtta-Vuori et al., 2008; Reineke et al., 2018). Accumulation of fatty acids in lipid droplets was measured as an increase of fluorescent intensity of a Bodipy-fatty acid dye. Fluorescence intensity was quantified inside lipid droplet area in 30 cells. To reduce the background staining, we recommend doing chase experiment, by incubating with Bodipy C12 followed by washing and 15 min chase (see Supplementary Figure 2E). Bodipy dye was added 15 min prior to the imaging unless indicated differently.

### Antibodies

We used the following reagents to detect proteins: monoclonal anti-G3BP (Sigma-Aldrich WH0010146M1), polyclonal anti-TIA1 produced in rabbit (Sigma-Aldrich SAB4301803), anti-GAPDH (sc-47724, Santa Cruz Biotechnology), anti-PPARA (sc-398394, Santa Cruz Biotechnology), anti-PPARG (sc-7273X, Santa Cruz Biotechnology), anti-PPARD (sc-74517, Santa Cruz Biotechnology), antiphospho-4EBP (Ser65, sc-293124), and anti-4EBP (sc-9977, Santa Cruz Biotechnology).

Secondary antibodies for immunofluorescence: anti-rabbit IgG Cy3-conjugated (Sigma-Aldrich C2306), anti-mouse IgG Cy3 conjugated (Sigma-Aldrich C2181), and anti-rabbit IgG Cy5 conjugated (Invitrogen A10523).

### Chemicals

BODIPY™ 558/568 C12 (4,4-difluoro-5-(2-thienyl)-4-bora-3a,4a-diaza-s-indacene-3-dodecanoic acid, Thermo Fischer Scientific), Hoechst (Sigma), sodium arsenite (Fischer Chemical), cycloheximide (Sigma), BODIPY™ 493/503 (ThermoFischer Scientific, D3922), Rosiglitazone (Sigma), Clofibrate (Sigma), GW501516 (Sigma), 2-bromopalmitic acid (Sigma), streptavidin-HRP (Thermo Scientific), fatty acid-free BSA (PAN Biotech), DMEM (PAN Biotech), FBS (PAN Biotech), PBS (PAN Biotech), methanol (Roth), chlorophorm (Sigma), aprotinin (Roth), leupeptin (Roth), phenylmethylsulfonyl fluoride (PMSF, Sigma), Fasnall (Sigma), and kinase screening library (*10505*, Cayman Chemical).

### CRISPR/Cas9

Knockout and endogenously tagged cell lines were constructed using CRISPR/Cas9 protocol and plasmids described in Ran et al. (Targett-Adams et al., 2003). Knockout cell lines were verified by western blotting. Genomic DNA was sequenced to verify disrupted region in knockout. CRISPR specificity was profiled using Digenome-Seq web tool (http://www.rgenome.net/cas-offinder/) (Ran et al., 2013). Off targets were not found. The following target sequences are used to modify genomic DNA: knockout of PPARA-CACAACCAGCACCATCTGGTCGCGA.

### Plasmid Construction

All plasmids were constructed using *Escherichia coli* strain DH5α. Plasmids used in this study are summarized in Table 1. We used px459 plasmid to clone CRISPR/Cas9 constructs for gene knockout. pSpCas9(BB)-2A-Puro (PX459) V2.0 was a gift from Feng Zhang (Addgene plasmid #62988; http://n2t.net/addgene:62988; RRID:Addgene_62988) (Targett-Adams et al., 2003).

**Table 1:** Plasmids used in this study.

**Plasmid name**	**Source**
Px459-PPAR-KO-gRNA	This study
pRFP-G3BP	Kaganovich Lab

### Microscopy

For live cell imaging we used four-well microscope glass-bottom plates (IBIDI) or Cellview cell culture dish (Greiner Bio One). Plates were coated with Concanavalin A (Sigma) for live cell imaging of yeast. Confocal images and movies were acquired using a dual point-scanning Nikon A1R-si microscope equipped with a PInano Piezo stage (MCL), temperature and CO_2_ incubator, using a ×60 PlanApo VC oil objective NA 1.40. We used 406, 488, 561, and 640 nm laser (Coherent, OBIS). Movies for kymographs were acquired in resonant-scanning mode. Image processing was performed using NIS-Elements software.

### Statistics and Data Analysis

Three or more independent experiments were performed to obtain the data. *P*-values were calculated by two-tailed Student *t*-test or one-way ANOVA for samples with *N* > 10 following normal distribution. Normal distribution of the data was verified using Shapiro-Wilk test, and the equality of variances was verified by Levene's test. Mann-Whitney or Kruskal-Wallis tests were used for experiments with < 5 samples or when samples did not follow a normal distribution. The sample sizes were not predetermined. Scatter plots were generated using Matplotlib (Hunter, 2007; Bae et al., 2014).

## Data Availability Statement

The raw data supporting the conclusions of this article will be made available by the authors, without undue reservation.

## Author Contributions

All aspects of the work comprising the manuscript were carried out jointly by DK and TA.

## Conflict of Interest

The authors declare that the research was conducted in the absence of any commercial or financial relationships that could be construed as a potential conflict of interest.

## Supplementary Material

Supplementary Figure 1 Inhibitor screen for fatty acid accumulation and SG formation. Cells expressing PABPC1-DDR2 were seeded on a 96-well glass-bottom plates and grown to 80–90% confluency. One hundred thirty-six inhibitors (100 μM) were added to the media for 1 h, and fatty acid dye (Bodipy-C12, Red, 1 μM) was added 30 min prior to the experiment. Cells were visualized by confocal microscopy; inclusion formation and fatty acid accumulation was assessed. Confocal images of all the samples are shown, refer to Figure 4C for zoomed in images. “*” fluorescence from the inhibitor.

Supplementary Figure 2 **(A)** Quantification of SG formation and LD accumulation during Fasnall treatment (corresponding to Figure 2B). Graph shows percentage of cells with SGs in the population and LD fluorescence intensity, mean ± SD. Pearson correlation coefficient (*r*) is 0.99. **(B,C)** Confocal microscopy of Bodipy and Bodipy-C12 costaining with and without (bottom row) washing. Intensity profile through the LD is shown **(B)**. **(D)** Confocal microscopy of HEK293T cell incubated with arsenite (200 μM) or arsenite and cycloheximide (10 μg/ml) for 90 min. SG marker G3BP is detected with an antibody, Bodipy, 1 μM was added 15 min prior to the imaging. Representative confocal planes are shown; scale bar, 5 μm. Graph shows a number of LDs per cell, mean ± SD. **(E)** Confocal microscopy of G3BP overexpression-induced SGs in HEK293T cells transfected with G3BP and stained with anti-TIA antibody. Representative confocal planes are shown; scale bar, 5 μm. **(F,G)** Quantification of the clustering, whole cell intensity, and the size of the LD in U2OS (WT and G3BP1/2 KO) cells during arsenite treatment, refer to Figure 2J, mean ± SD, **P* < 0.01.

Supplementary Figure 3 **(A)** Confocal microscopy of SG and LD formation during oleic acid (OA) treatment. HEK293T cells were treated with the indicated amounts of OA for 24 h before the imaging. SGs are visualized with an endogenous PABPC1-DDR2. LDs are stained with Bodipy. **(B)** PPAR activation leads to mTOR inactivation. Cells were incubated with PPAR activators (rosiglitazone, clofibrate, and GW501516, 100 μM) for 3 h. mTOR activity was assessed by antiphopho-4EBP and microscopy of cells during PPAR activators treatment; scale bar, 5 μm. **(C)** Immunofluorescence showing SG marker localization to the PABPC1-DDR2 inclusions during PPAR activation. PABPC1-DDR2 cells were treated with clofibrate, rosiglitazone, and GW501516 (100 μM) for 1 h. Cells were fixed and stained with anti-TIA1 and anti-G3BP antibodies. Hoechst (10 μg/ml) was used to stain the nucleus 15 min prior to imaging. Confocal planes are shown; scale bar 5 μm. **(D)** SG formation timeline during PPAR activation showing fatty acid accumulation. Cells expressing CRISPR/Cas9-tagged PABPC1-DDR2 were incubated with rosiglitazone, clofibrate, and GW501516 (100 μM) for indicated amounts of time, LD dye (Bodipy-C12, 1 μM) was added 30 min prior to the imaging together with Hoechst (10 μg/ml). Representative confocal images are shown. Arrows indicate SGs; scale bar, 5 μm. **(E)** SG formation in SHSY-5Y cells. Cells were treated with arsenite (100 μM) or 2-BP (200 μM) for 1 h and fixed. Stress granules marker (G3BP and TIA1) were visualized using immunostaining. Representative confocal planes are shown; arrowheads indicate SGs; scale bar, 5 μm.
