# Supplementary figures and images for: Small Molecule Screen Reveals Joint Regulation of Stress Granule Formation and Lipid Droplet Biogenesis
Source: Front Cell Dev Biol. 2021 Apr 16;8:606111. doi: 10.3389/fcell.2020.606111 (PMC8105174; doi:10.3389/fcell.2020.606111)

Figure S1

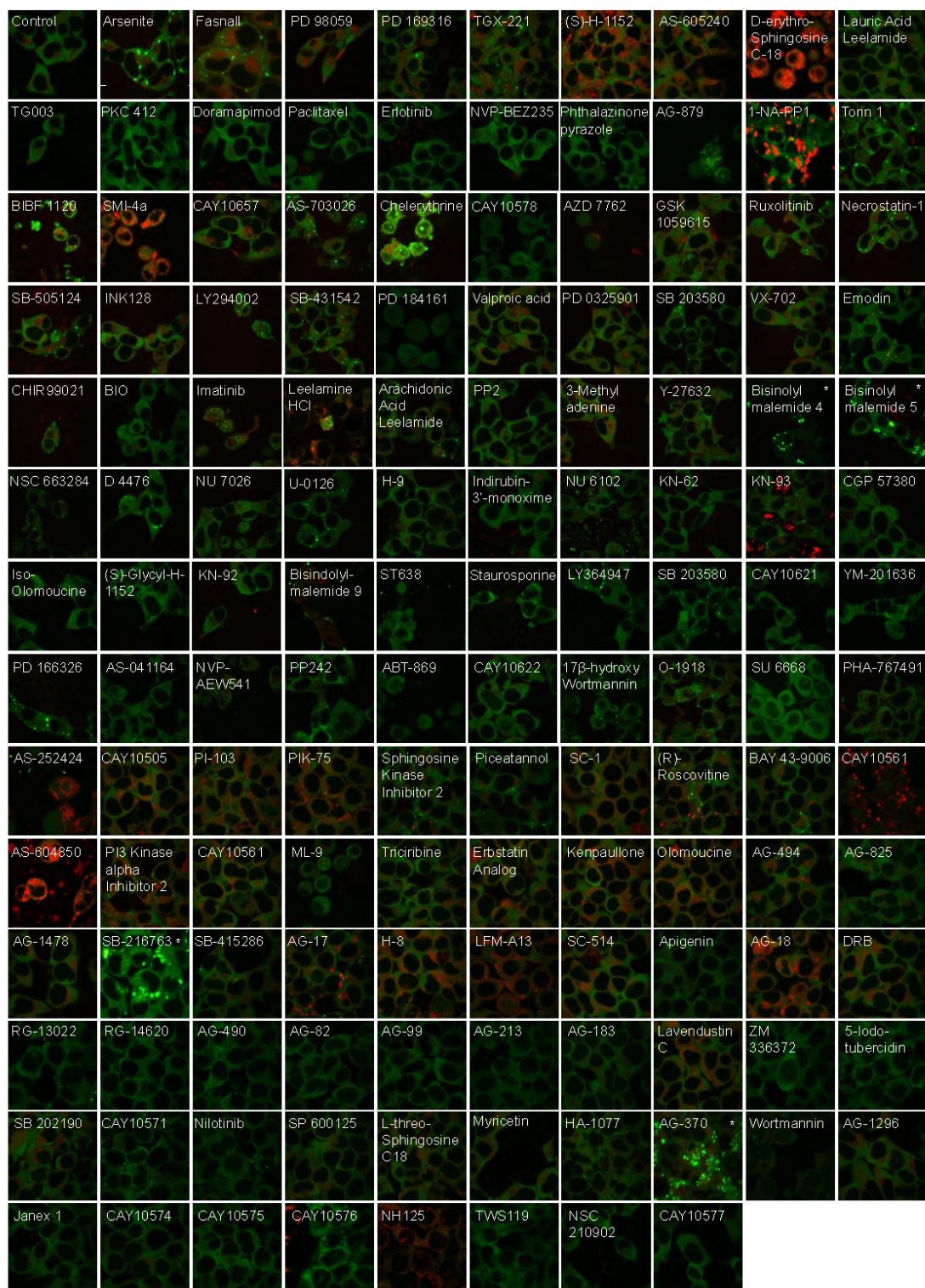

Figure S2

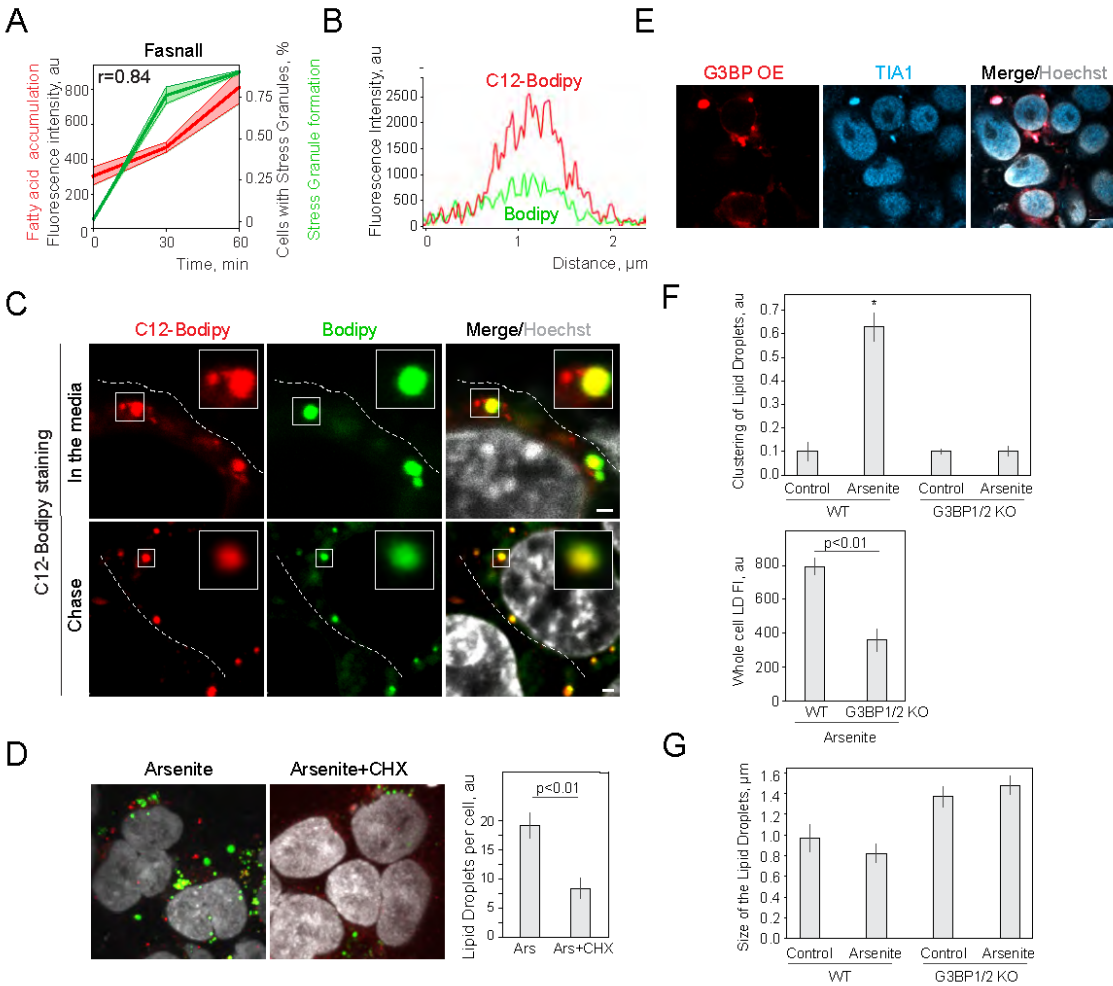

Amen and Kaganovich, Figure S3

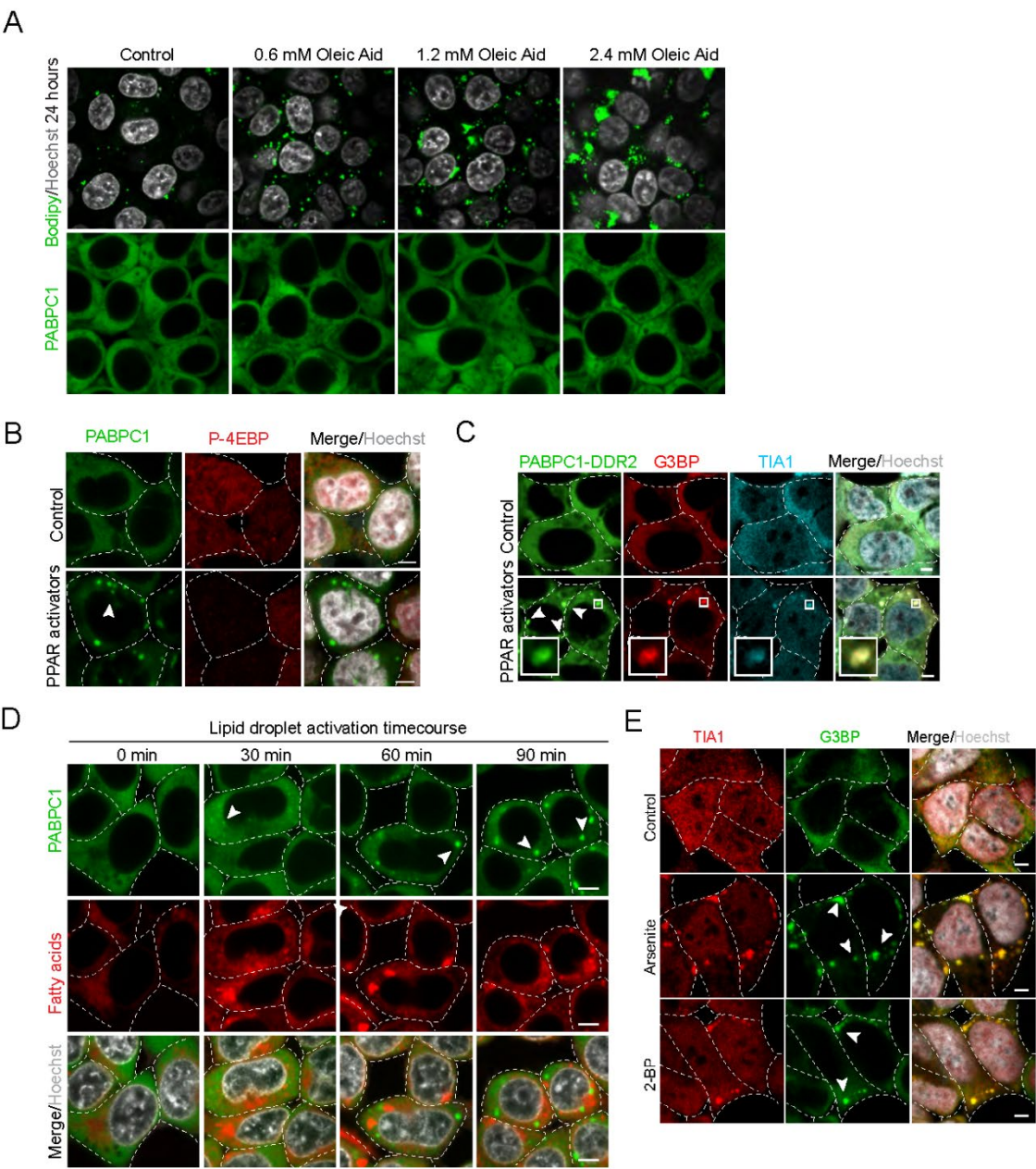

Supplement: Supplementary Figure 1 — Inhibitor screen for fatty acid accumulation and SG formation. Cells expressing PABPC1-DDR2 were seeded on a 96-well glass-bottom plates and grown to 80–90% confluency. One hundred thirty-six inhibitors (100 μM) were added to the media for 1 h, and fatty acid dye (Bodipy-C12, Red, 1 μM) was added 30 min prior to the experiment. Cells were visualized by confocal microscopy; inclusion formation and fatty acid accumulation was assessed. Confocal images of all the samples are shown, refer to Figure 4C for zoomed in images. “*” fluorescence from the inhibitor. [file Data_Sheet_1.pdf]
